# Disabilities and Handicaps of Patients with Laron Syndrome

**DOI:** 10.3390/children12091271

**Published:** 2025-09-22

**Authors:** Zvi Laron

**Affiliations:** Schneider Children’s Medical Center, Petah Tikva, Faculty of Medical & Health Sciences, Tel Aviv University, Tel Aviv-Yafo 6997801, Israel; laronz@clalit.org.il

**Keywords:** Laron Syndrome, GH insensitivity, GH-Receptor, IGF-I deficiency, disabilities, dwarfism, obesity, diabetes, cardiovascular disease

## Abstract

**Background**: Laron Syndrome (LS) is a rare hereditary form of dwarfism occurring, with few exceptions, in Jewish, Muslim, and Asian populations or their descendants spread over all continents. It is caused by deletions or mutations in the GH-Receptor gene, resulting in high serum levels of a structurally and biologically normal, but inactive GH and low-to-undetectable IGF-I. **Aim**: To summarize the disabilities and handicaps observed in patients with LS, from infancy through adult age. **Results**: Diagnosing, treating and following a cohort of 76 patients with LS (in many cases from infancy into adult age) enabled our department to study not only their growth and social achievements, but also the difficulties these patients encounter in life. The longstanding IGF-I deficiency caused somatic and biochemical changes which led to disabilities starting in infancy and becoming more severe with advancing age. The most serious symptoms LS patients have are dwarfism, progressive obesity, diabetes, fatty liver, cardiovascular disease, and neurological and orthopedic problems, leading to difficulties in vocational training, occupation, and social life, all lowering the Quality of Life (QoL) of these patients. **Conclusions**: Early initiation of IGF-I replacement treatment in patients with Laron Syndrome prevents and reverses some of the symptoms associated with longstanding IGF-I deficiency.

## 1. Introduction

Laron Syndrome (LS—OMIM #262500, MedDRA #10082851, SCID #38196001, ICD-10 E34.3, GARD 6859, UMLS C0271568, MeSH D046150, Orpha 633) or GH insensitivity is a rare disease of growth failure falling in the category of “Orphan Diseases”. It constitutes the best characterized entity of congenital IGF-I deficiency [1]. LS was first described in 1966 [2] and 1968 [3] in Yemenite Jews. Following these reports, more diagnoses were established in children originating from the Mediterranean, the Middle East, Asia, or South and Central America [4,5]. Despite their resemblance with genetic isolated growth hormone (GH) deficiency [6], these patients had high serum GH and low-to-undetectable insulin-like growth factor I (IGF-I) which did not respond to exogenous GH administration [7,8] demonstrating GH insensitivity [9,10]. By analyzing liver membranes from two LS patients, it was found that the resistance to GH is due to the inability of the GH molecule to bind to its receptor [11]. Chromatography and PCR methods revealed that the underlying pathology is caused by deletions or mutations in the GH-Receptor gene [12,13]. The great majority of the defects were found to be in the extracellular domain of the GH-Receptor gene [14]. The analysis of our cohort, all comprising consanguineous families, led to the conclusion that LS is genetically transmitted by a fully penetrant autosomal recessive mechanism [15]. It is noteworthy that only patients who are homozygous or double heterozygotes for GH-R defects express the full characteristics of the syndrome. The resemblance of the bone X-rays of our patients with the skeleton of an 18,000-year-old female with dwarfism on the Island of Flores led us to hypothesize that the founder gene originated in Indonesia [16]. For over 50 years we have followed a large cohort of LS patients [17], many of whom we have followed from early childhood into adult age. This enabled us to study not only the clinical and biochemical consequences of IGF-I deficiency and IGF-I replacement in LS patients but also their adjustment to society at various age levels.

The aim of this report is to describe the disabilities and handicaps observed in our large cohort of patients with Laron Syndrome.

## 2. Subject and Methods

The data were extracted from the Medical Records of the Endocrinology and Diabetes Research Unit of the Schneider Children’s Medical Center. Sixty-nine patients and their families lived in Israel and seven were referred from other countries.

Only patients with a proven and documented diagnosis of Laron Syndrome, i.e., high serum GH, low serum IGF-I, and lack of response upon GH administration with or without genetic analysis were included in the study.

The study was approved by the Rabin Medical Center Ethics Committee (RMC: 619-22).

## 3. Results

Below, we have listed the main disabilities and symptoms observed in LS patients studied by us.

### 3.1. Growth and Development

**Figure 1:**
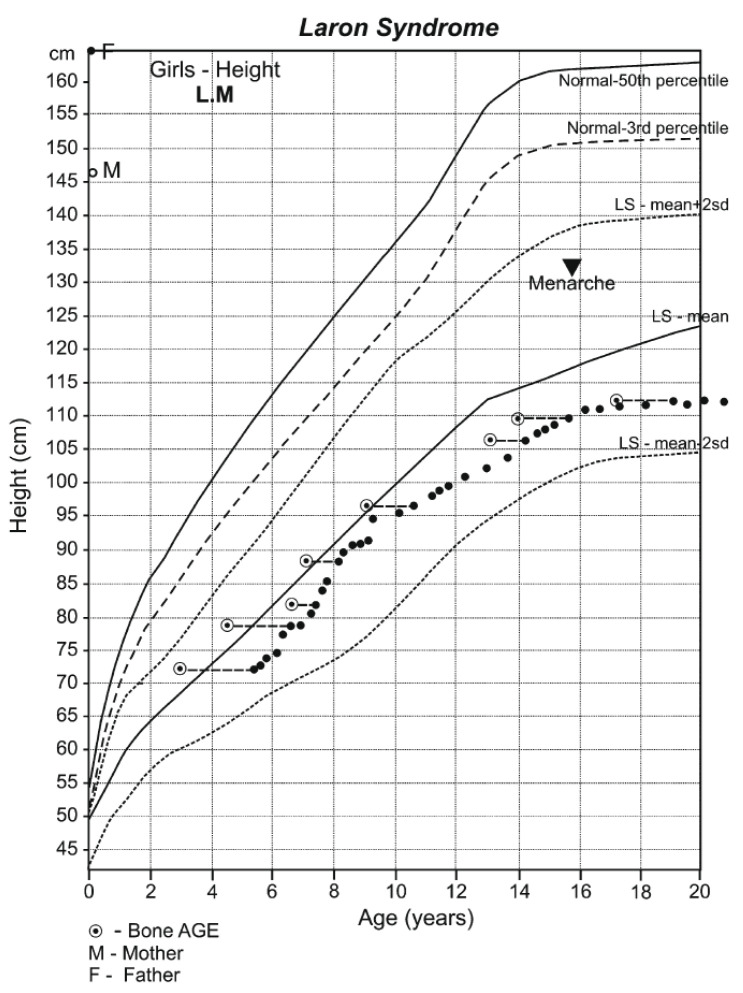
Growth pattern of an untreated girl with Laron Syndrome. Drawn on the Laron Syndrome-specific growth chart (Laron Z et al. *Arch Dis Child.* **1993**, 68: 768-70) [31]; reproduced with permission from Laron Z, Kopchivk JJ. *Laron Syndrome—from Man to Mouse.* Heidelberg, Springer—Verlag 2011 [32].

**Figure 2:**
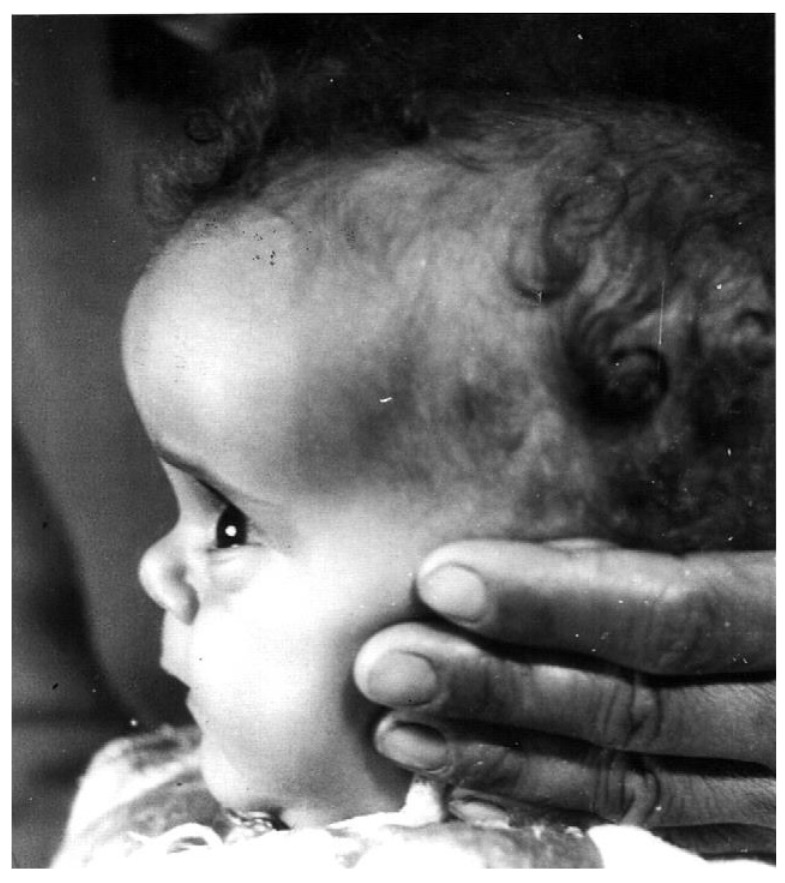
Characteristic features of a 1-year-old girl with Laron Syndrome. Note the protruding forehead, saddle nose, and sparse hair [3,6]. Reproduced with permission from: Laron Z, Kopchivk JJ. *Laron Syndrome—from Man to Mouse*. Heidelberg, Springer: Verlag 2011 [33].

Dwarfism is present at birth (42–46 cm) and progresses until closure of the long bone epiphysis, leading to an adult height of 116–142 cm in males and 108–136 cm in females [18] (Figure 1). The upper/lower body ratio reveals short lower limbs compared to the trunk. The head circumference (i.e., brain volume) is smaller than normal [19], and there is underdevelopment of the facial bones [20,21] which leads to a protruding forehead, saddle nose, sunset look, and sparse hair resulting in a characteristic abnormal facies from infancy [3,6] (Figure 2). The hands and feet are small (acromicria) [22,23], resulting in difficulties finding appropriate clothing. Some infants are obliged to wear doll shoes [24] due to an inability to find small enough shoes. These unusual features, together with the dwarfism, draw attention, causing stress to parents and antagonism in surrounding people. Due to the abnormal structure of the cranium, LS patients have a retarded growth of the ocular globe [25,26] causing myopia and the need for spectacles. The retinal vascularization is reduced [27] and with advancing age, a minority of patients develop glaucoma. Some are born with strabismus or cataracts; all these issues impair vision. LS patients also develop a neurosensory hearing defect [28]. The development, size, morphology, and composition of the teeth are defective [29] and orthodontic treatment is required. Due to the immaturity of the carbohydrate system, infants and young children suffer from hypoglycemia and seizures [30]. This feature is reversed with the increase in the degree of the adipose tissue during puberty.

### 3.2. School Age

To adjust to the dwarfism, low benches and tables are needed. Deficiencies in protein metabolism, such as an abnormal amino acid pattern [34] and low procollagen levels [35], lead to underdevelopment of the muscular system and weakness [36,37] which impedes mobility, participation in competitive sport activities, and carrying heavy school bags. Children with LS also find it difficult to manage the steps when getting on and off regular school buses. Due to their abnormal appearance, obesity, and size, LS children are often bullied by their classmates.

### 3.3. Sexual Development and Puberty

Penis size, testicular volume, and female genitalia are small at all ages [38,39] but do not impair sexual activity and reproduction later in life [17,40]. Puberty is delayed in both sexes [41] which differentiates patients with LS from their peers and causes feelings of inferiority.

### 3.4. Adult Life

**Figure 3:**
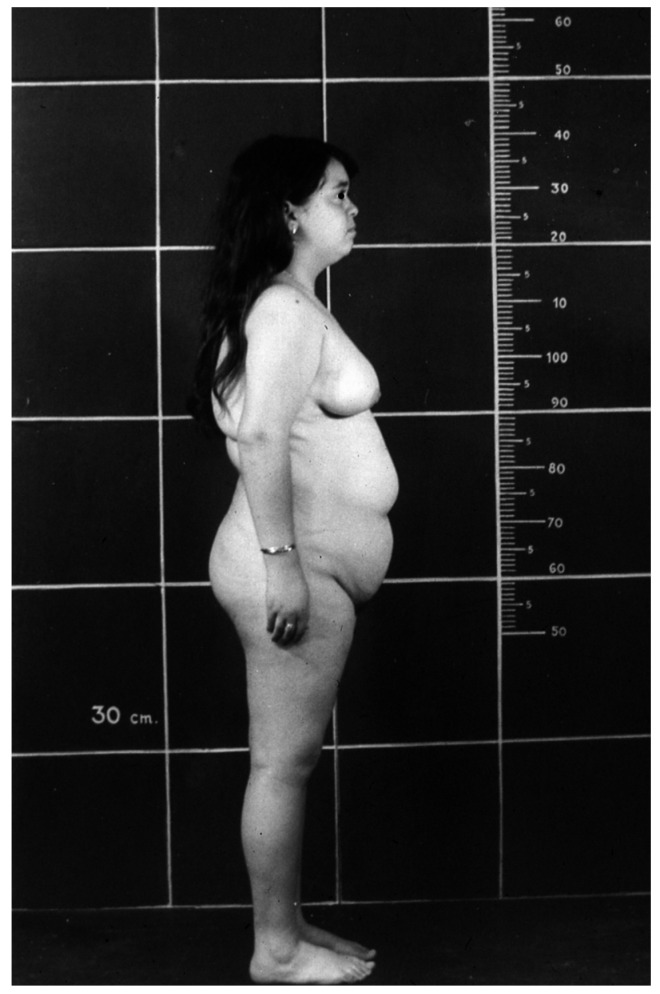
Obesity in a 16-year-old girl with Laron Syndrome. Reproduced with permission from Laron Z, Kopchivk JJ. *Laron Syndrome—from Man to Mouse.* Heidelberg, Springer: Verlag 2011 [33].

The progressive obesity [33] (Figure 3), independent of food intake [42], impedes a normal life routine and causes clinical and biochemical complications, such as insulin resistance, glucose intolerance, diabetes, hyperlipidemia, fatty liver, and cardiovascular disease, all characteristics of the “Metabolic Syndrome” [43].

### 3.5. Limitation in Mobility

Two of our patients and 25% of the Ecuadorian patients have congenital dislocation of the hip or Perthes Disease [44]. If not treated, together with the weakness of the muscular system and marked obesity, serious walking impairment may occur. A reduced left heart output and reduced lung function also impair exercise capacity [45].

### 3.6. Neurological Abnormalities

Using X-rays, CT, and MRI examinations of the skull and brain, we found a series of structural pathologies in the central nervous system (CNS) leading to neurologic deficits [46]. Among these are underdeveloped sinuses, degenerative changes of the white matter with occasional cerebellar atrophy, and progressive development of spinal stenosis [47]. An unusual brain lesion was found in one of our patients [48]. A similar lesion was reported in an LS patient from Mexico [49]. Both were adult female patients who died suddenly. Few patients had focal epilepsy [50] and due to the reduced dimensions of the larynx and obesity, LS patients often suffer from sleep apnea [51,52].

### 3.7. Psycho-Social Aspects

Psychological studies of LS children and adults revealed that some of the children present a low score on the Wechsler and Bender tests [53,54]. The low stature and academic limitations of some patients cause difficulties with regard to vocational training and finding occupations [17]. A minority of the patients we have followed achieved academic degrees, with one achieving a PhD degree. Some of the patients with healthy partners are married and have children. Others have difficulties finding partners.

**Figure 4:**
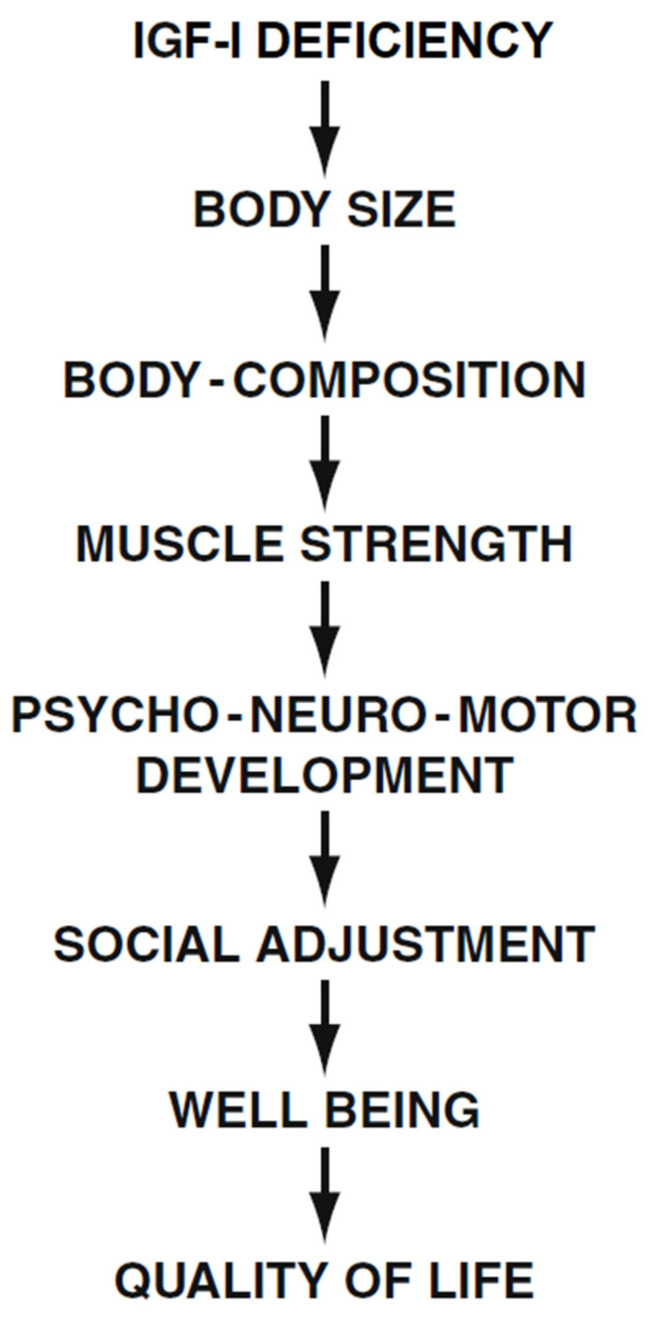
Effect of long-term IGF-I deficiency on social adjustment and Quality of Life (QoL) in patients with Laron Syndrome. Reproduced with permission from Laron Z, Kopchivk JJ. *Laron Syndrome—from Man to Mouse.* Heidelberg, Springer: Verlag 2011 [32].

The many symptoms, social limitations, and difficulties these patients face at any age are reflected in the emotional states, disappointment, and even depression reported by most LS patients. Raising a child with Laron Syndrome and being a patient with many limitations causes great psychological problems in the family and in the patient themselves, with a negative impact on Quality of Life [55] (Figure 4). The fact that patients with LS are protected from developing malignancies [56,57] was not found to affect their QoL.

### 3.8. Treatment

The only treatment for LS is replacement of the genetic deficiency of IGF-I. IGF-I is a peptide which needs to be injected daily, preferably once a day [58,59]. Administering it twice or in greater doses causes unnecessary and unpleasant adverse effects [60]. IGF-I treatment accelerates linear growth [52,58,61,62] and growth of the head circumference (i.e., brain size) [19]. Even when the treatment is continuous, most children do not reach normal height. Whereas short-term treatment reduces adiposity, long-term administration of IGF-I stimulates the development of obesity [63]. As IGF-I treatment is currently approved only for children, to stimulate growth, the symptoms associated with late complications are not prevented. In a short clinical trial of IGF-I treatment of adult patients with LS, we found that IGF-I had beneficial effects such as lowering blood insulin, cholesterol [64] and serum Lp (a) [65], and that it improved left heart ventricular function [66].

## 4. Discussion

Laron Syndrome is a rare disease but a unique model for understanding the role of IGF-I in the process of growth and body composition [1,67]. After its discovery in 1966 [2], more and more patients were referred to our clinic and reports from other countries were published in the scientific literature. Long-term follow up was reported only by two clinics with many patients (i.e., in Ecuador [68] and Israel [17]). As our patients lived in a small country with easy access to a multidisciplinary medical team and provision of complete insurance for all medical expenses, we had a better opportunity to follow our patients than our Ecuadorian colleagues, whose patients are dispersed in locations far from the clinic, meaning that some of their examinations had to be performed in the USA. Comparing the findings between the two cohorts, it is evident that the patients in Ecuador had all the complications, disabilities, and symptoms found in our LS patients [69,70], including morphological changes in the brain structure [71]. It is noteworthy that the patients in Ecuador showed a high incidence of cardiac disease (27%), stroke, alcoholism, and a high incidence of accidents (20%) [69]. It thus seems that independent of the local conditions, the disease led to a reduced QoL.

### Future Perspectives

All of the disabilities and handicaps of patients with LS are inherent to the genetic IGF-I deficiency. Height can be improved by early initiation of IGF-I replacement treatment. Even if normal height cannot be achieved, social adjustment is improved. The recently introduced weight reduction treatments using GLP-1 receptor agonists or gastric sleeve operation started by a few of our patients reduced obesity and reduced some of its biochemical complications. The marked loss of weight also had positive psychological effects. Unfortunately, both IGF-I and GLP-1-R antagonists are available to only a small number of LS patients due to regulatory and economic factors in some countries. The same is true for pregestational diagnosis and implantation of only healthy embryos in an attempt to prevent new patients with LS in families at risk. Gene therapy may also be a possible future treatment [72].

## 5. Conclusions

Patients with Laron Syndrome due to defects in the GH-R gene and resulting lifelong IGF-I deficiency suffer from many disabilities starting in infancy, comprising biochemical and clinical changes in all body systems. These abnormalities cause many physical symptoms and psycho-social problems, which are only partially alleviated by IGF-I replacement treatment.

## Data Availability

The patients or parents approved the use of academic data for academic purposes.
